# Human retinal pigment epithelial cells

**DOI:** 10.1111/cpr.13153

**Published:** 2021-11-12

**Authors:** Lei Wang, Jiani Cao, Yaojin Peng, Boqiang Fu, Zibing Jin, Yuntao Hu, Wei Wu, Andy peng Xiang, Shijun Hu, Junying Yu, Yu Zhang, Jun Wei, Yong Zhang, Qiyuan Li, Jiaxi Zhou, Peijun Zhai, Huanxin Zhu, Lingmin Liang, Aijin Ma, Glyn Stacey, Tongbiao Zhao, Jie Hao

**Affiliations:** ^1^ National Stem Cell Resource Center State Key Laboratory of Stem Cell and Reproductive Biology Institute of Zoology Institute for Stem Cell and Regeneration Chinese Academy of Sciences University of Chinese Academy of Sciences Beijing China; ^2^ Beijing Institute for Stem Cell and Regenerative Medicine Beijing China; ^3^ Chinese Society for Stem Cell Research Shanghai China; ^4^ China National Institute of Metrology Beijing China; ^5^ Beijing Institute of Ophthalmology Beijing Tongren Hospital Capital Medical University Beijing China; ^6^ Beijing Tsinghua Changgung Hospital School of Clinical Medicine Tsinghua University Beijing China; ^7^ Department of ophthalmology Chinese PLA General Hospital Beijing China; ^8^ Center for Stem Cell Biology and Tissue Engineering Key Laboratory for Stem Cells and Tissue Engineering Ministry of Education Sun Yat‐Sen University Guangzhou China; ^9^ Department of Cardiovascular Surgery of the First Affiliated Hospital & Institute for Cardiovascular Science Collaborative Innovation Center of Hematology State Key Laboratory of Radiation Medicine and Protection Medical College Soochow University Suzhou China; ^10^ Nuwacell Biotechnology Co., Ltd Hefei City China; ^11^ Zephyrm Biotechnologies Co., Ltd Beijing China; ^12^ HHLIFE Company Inc Shenzhen China; ^13^ China National GeneBank Shenzhen China; ^14^ Institute of Hematology and Blood Diseases Hospital Chinese Academy of Medical Sciences & Peking Union Medical College Beijing China; ^15^ China National Accreditation Service for Conformity Assessment Beijing China; ^16^ Beijing Technology and Business University Beijing China

**Keywords:** applications, human retinal pigment epithelial cells, quality control, standard

## Abstract

'Human retinal pigment epithelial cells' is the first set of guidelines on human retinal pigment epithelial cells in China, jointly drafted and agreed upon by experts from the Chinese Society for Stem Cell Research. This standard specifies technical requirements, test methods, inspection rules, instructions for usage, labelling requirements, packaging requirements, storage requirements and transportation requirements and waste disposal requirements for human retinal pigment epithelial cells, which is applicable to quality control during the process of manufacturing and testing of human retinal pigment epithelial cells. It was originally released by the Chinese Society for Cell Biology on 9 January 2021. We hope that publication of these guidelines will promote institutional establishment, acceptance and execution of proper protocols and accelerate the international standardization of human retinal pigment epithelial cells for applications.

## 1 SCOPE

This document specifies the technical requirements for human retinal pigment epithelial cells, and the requirements for the test methods, instructions for use, labelling, packaging, storage, transportation and waste disposal.

This standard is applicable for the production and testing of human retinal pigment epithelial cells.

Note: Human retinal pigment epithelial cells include primary sources and stem cell differentiation sources.

## 2 NORMATIVE REFERENCES

The following content constitute indispensable articles of this standard through normative reference. For dated references, only the edition cited applies. For undated references, only the latest edition (including all amendments) applies.

*GB/T* *6682 Water for analytical laboratory use – Specification and test methods*.

*Ws* *293 Diagnosis for HIV / AIDS*.

*Ws* *213 Diagnosis of hepatitis c*.

*Ws* *273 Diagnosis for syphilis*.

*Pharmacopoeia* *of the People's Republic of China*.

*National* *Guide to Clinical Laboratory Procedures*.

## 3 TERMS AND DEFINITIONS

The following terms and definitions apply to this document.

### 3.1 Human retinal pigment epithelial cell

The hexagonal monolayer epithelial cells containing pigment granules, which can be acquired by primary isolation from human retina, differentiation from stem cell and trans‐differentiation from other somatic cells.

## 4 ABBREVIATIONS

The following abbreviations apply to this document.

Best1: bestrophin 1;

EBV: Epstein‐Barr virus;

HBV: Hepatitis B virus;

HCMV: Human cytomegalovirus;

HCV: Hepatitis C virus;

HIV: Human immunodeficiency virus;

HTLV: Human T‐lymphotropic virus;

MITF: Microphthalmia‐associated transcription factor;

OTX2: Orthodenticle homeobox 2;

PEDF: Pigment epithelium‐derived factor;

RPE65: Retinal pigment epithelial cell 65 protein;

STR: Short tandem repeats;

TP: Treponema pallidum;

VEGF: Vascular endothelial growth factor;

ZO‐1: Zonula occludens‐1;

## 5 TECHNICAL REQUIREMENTS

### 5.1 Source materials and ancillary materials

5.1.1. The collection of human biological source material (source material) shall comply with the domestically recognized ethics and local laws and regulations.

5.1.2. Written and valid informed consent shall be signed by the donor. The content of informed consent shall include but not limited to the research purpose, potential research and clinical application under appropriate conditions feedback on unexpected discoveries potential commercial value and other issues affiliated to the research. Mechanisms for protecting the personal data and privacy of donors shall be established.

5.1.3. Organizations performed cell research and/or production shall establish and implement cell donor evaluation standards, and establish collection methods, transportation standards, handover standards and storage standards to ensure the safety of donors and cells. The source material shall be accompanied with detailed documentation on the acquisition methods and donor information, including but not limited to the donor's general information, past medical history and family history. The information of donor's blood type (e.g., ABO, Rh) and human leucocyte antigen (HLA) alleles should be documented as necessary.

5.1.4. The origin of cells shall be traceable by referring to the relevant informed consent and/or their genome and functional data.

5.1.5. Ancillary materials such as culture medium and growth factors shall meet the corresponding quality control requirements. The ancillary materials can be inspected and laboratory tested if necessary.

5.1.6. When using animal serum, they shall be free of contamination by viruses of animal origin. Serum from animals in geographical regions with prion epidemics (e.g., bovine spongiform encephalopathy) shall be prohibited.

5.1.7. If human blood components are used in the culture medium, including but not limited to albumin, transferrin and various cytokines, the source, batch number and quality verification reports shall be provided. State‐approved products shall be used as much as possible.

5.1.8. The donors shall be screened for HIV, HBV, HCV, HTLV, EBV, HCMV and TP, and the results shall be documented.

### 5.2 Critical quality attributes

#### 5.2.1 Cell morphology

Pigment can be seen in the cells. Under the condition of monolayer adherent growth, the cells are in close contact and polygonal.

#### 5.2.2 Chromosome karyotype

The normal karyotype shall be 46, XY or 46, XX.

#### 5.2.3 Cell survival rate

Shall be ≥90% before cryopreservation, and ≥50% post‐thaw.

#### 5.2.4 Cell marker protein

The expression of ZO‐1 shall be ≥ 70% of the cell population. The expression of at least any three of the cell markers RPE 65, OTX2, MITF and BEST1 shall be ≥ 70% of the cell population.

#### 5.2.5 Secretory function

Shall have the capacity of secreting PEDF and VEGF.

#### 5.2.6 Microorganisms

Shall be negative for fungi, bacteria, mycoplasma, HIV, HBV, HCV, HTLV, HEBV, HCMV and TP.

### 5.3 Process control

#### 5.3.1 Expansion

5.3.1.1. During the process of cell expansion, cross‐contamination and mislabelling of cell lines shall be avoided, and risk mitigation measures shall be established.

5.3.1.2. During the process of cell expansion, the passage and the name of the cells shall be clearly specified, and the operation date, culture conditions, operators' names or initials shall be indicated.

#### 5.3.2 Differentiation

5.3.2.1. During cell differentiation, the starter cells, equipment, culture conditions and operation procedures shall be defined and documented. The standard operating procedure for cell differentiation shall be established for reproducibility.

5.3.2.2. The differentiated cells from stem cells shall be clearly defined using characteristics, including but not limited to morphology, marker gene expression and functionality.

Note: This part is applicable for stem cell derived human retinal pigment epithelial cells.

#### 5.3.3 Cryopreservation

5.3.3.1. Cryopreserved cells shall be clearly labelled with the cell line name, culture conditions, passage number, operator name and cryopreservation date. Cryopreserved cells shall have the same unique identification used during the process of harvesting, separation, expansion, etc.

5.3.3.2. The cryopreservation procedure shall follow the known principles of cryopreservation of mammalian cells and shall be recorded according to the relevant regulations.

#### 5.3.4 Resuscitation

5.3.4.1. The resuscitation process shall be as rapid as possible to ensure the optimal viability and biological activity of cells.

5.3.4.2. Cell line information including but not limited to the cell line name, passage number, culture conditions, operator name or initials, resuscitation date and time shall be documented and recorded.

#### 5.3.5 Cell STR identification

The STR signature of hRPEs shall be consistent with that of donor cells.

## 6 TEST METHODS

### 6.1 Cell morphology

Under the condition of two‐dimensional adherent culture in vitro, the cells shall be cultured for at least 10 days and observed by inverted microscope.

### 6.2 Chromosome karyotype

The method in the *Pharmacopoeia of the People's Republic of China* shall be followed.

### 6.3 Cell survival rate

The method in Appendix A shall be followed.

### 6.4 Cell markers

The method in Appendix B shall be followed.

### 6.5 Secretory function

The method in Appendix C shall be followed.

### 6.6 Microorganisms

#### 6.6.1 Fungi

6.6.1. The method in *Pharmacopoeia of the People's Republic of China* shall be followed.

#### 6.6.2 Bacteria

6.6.2. The method in *Pharmacopoeia of the People's Republic of China* shall be followed.

#### 6.6.3 Mycoplasma

The method in *Pharmacopoeia of the People's Republic of China* shall be followed.

#### 6.6.4 HIV

The method in WS 293 shall be followed.

#### 6.6.5 HBV

The method in the National Guide to Clinical Laboratory Procedures shall be followed.

#### 6.6.6 HCV

According to WS 213 nucleic acid method.

#### 6.6.7 HTLV

The method in the National Guide to Clinical Laboratory Procedures shall be followed.

#### 6.6.8 EBV

The method in the National Guide to Clinical Laboratory Procedures shall be followed.

#### 6.6.9 HCMV

The method in the National Guide to Clinical Laboratory Procedures shall be followed.

#### 6.6.10 TP

The method in WS 293 shall be followed.

## 7 INSPECTION RULES

### 7.1 Sampling method

7.1.1. Cells produced from the same production cycle, same production line, same source, same passage and same method are considered to be the same batch.

7.1.2. Three smallest units of packaging shall be randomly sampled from the same batch.

### 7.2 Quality inspection and release

7.2.1. Each batch of products shall be subject to the qualify inspection before release, and inspection reports shall be attached.

7.2.2. The quality inspection items shall include all the attributes specified in 5.2.

### 7.3 Review inspection

Review inspection shall be performed by professional cytological testing institutions/laboratories as necessary.

### 7.4 Decision rules

7.4.1. Products that pass all requirements in 5.2 for the quality inspection for release are considered to be qualified. Products that fail to pass one or more requirements in 5.2 for the quality inspection for release are considered to be unqualified.

7.4.2. Products that pass all requirements in 5.2 for the quality review inspection are considered to be qualified. Products that fail to pass one or more requirements in 5.2 for the review inspection are considered to be unqualified.

## 8 INSTRUCTIONS FOR USAGE

The instructions for usage shall include but not limited to:

a. Product name;

b. Passage number;

c. Cell numbers;

d. Production date;

e. Lot number;

f. Production organization;

g. Storage conditions;

h. Shipping conditions;

i. Contact information;

j. Operation manual;

k. Execution standard number;

Note: According to what standards are the cells produced.

l. Manufacturing address;

Note: alternatively refers to the derivation laboratory.

m. Postal code;

n. Matters that need attention.

Endotoxin content can be marked according to user's requirement.

## 9 LABELS

The label shall include but not limited to:

a. Product name;

b. Passage number;

c. Cell number;

d. Lot number;

e. Production organization;

f. Production date.

## 10 PACKAGE, STORAGE AND TRANSPORTATION

### 10.1 Package

The appropriate materials and containers shall be selected to ensure maintenance of the primary quality attributes of human retinal pigment epithelial cells.

### 10.2 Storage

10.2.1. Principles and procedures for cell storage management shall be developed and followed, and detailed information shall be recorded, including but not limited to written applications to cell storage organizations, ethical review committee approval documents and cell line specific information.

10.2.2. The application for the use of cells shall be submitted by the user organization or individual and approved by the cell storage organization.

10.2.3. Cells stored in the cell bank shall meet the corresponding cell bank management requirements.

10.2.4. Shall be stored below‐130℃.

### 10.3 Transportation

10.3.1. According to the requirements of cell use, appropriate transport modes and conditions shall be selected to ensure cell biological characteristics, safety, stability and effectiveness.

10.3.2. Cell transport shall take the following factors into account, including but not limited to, cell characteristics, containers carrying cells, transport routes, transport conditions, transport equipment, modes of transport, transport risks and safeguards.

10.3.3. The management of transport conditions shall include but not limited to, the temperature range, oscillation, non‐pollution, equipment performance and packaging, etc.

10.3.4. The cell transportation shall be recorded, including but not limited to, the manner and conditions, path, time, personnel, address and cellular information of cell transport.

10.3.5. Cryopreserved cells should be transported in dry ice or below‐130 ℃, while non‐frozen cells should be transported at 2℃~8℃.

## 11 WASTE DISPOSAL

11.1. The waste generated during the production and testing of human retinal pigment epithelial cells shall be in accordance with the waste cell management documents, strict implementation of management standards and detailed records.

11.2. Unqualified cells, remaining discarded cells or donations in the research and production of human retinal pigment epithelial cells should be disposed of legally, properly and ethically.

## Supporting information

Supplementary Material
