# Supplementary material for: Human retinal pigment epithelial cells
Source: Cell Prolif. 2021 Nov 12;55(4):e13153. doi: 10.1111/cpr.13153 (PMC9055896; doi:10.1111/cpr.13153)
Supplement: Supplementary file 1 — Supplementary Material [file CPR-55-e13153-s001.docx]

**Appendix A**

**(Normative appendix)**

**Cell survival rate test (cell enumeration method)**

**A.1 Instruments and equipment**

A.1.1 Microscope.

A.1.2 Hemocytometer.

A.2 Reagents

Unless otherwise stated, all reagents used shall be analytical grade. The water used for testing shall be deionized water.

A.2.1 Phosphate buffered saline (PBS): pH 7.4.

A.2.2 Trypan blue solution.

**A.3 Testing protocol**

A.3.1 Preparation of cell suspension

Harvest and suspend the cells with appropriate volume of DPBS (A.2.1). The cells in the hemocytometer shall be 20–50 cells / mm^2^. Serial dilution is necessary if the number of cells exceeds 200 per hemocytomoter.

A.3.2 Trypan blue staining

Evenly mix the Trypan blue solution (A.2.2) with the cell suspension (A.3.1) at a volume ratio of 1:1.

A.3.3 Cell counting

Load the hemacytometer (A.1.2) with 10 μL of the trypan blue-labeled sample (A.3.2). Make sure the entire chamber is filled with the testing sample. Stand for 30 seconds, count the stained cells and the total number of cells respectively.

For the 16×25 counting chamber, use the four 1 mm^2^ medium squares at the top left, top right, bottom left, and bottom right of the chamber (i.e. 100 small squares) for counting.

For the 25×16 counting chamber, use the five 1 mm^2^ medium squares at the top left, top right, bottom left, bottom right, and center of the chamber (i.e. 80 small squares) d for counting.

When there are cells on the lines of the large square, only cells on the top line and left line of the large square can be counted (or alternatively only cells on the bottom line and right line).

**A.4 Calculation and analysis**

Cell viability is calculated according to equation (1):

*S*=（*M*－*D*）/*M*×100% (1)
In the equation:

*S*——*viability of cells*

*M——total number of cells*

*D——number of stained cells*

The viability of cells is the mean of two duplicate samples. Two independent cell viability tests shall be performed on the same sample. The mean value of two independent viability tests is recorded as the viability of cells.

**A.5 Accuracy**

The absolute difference value between the two independent tests, under the same conditions, shall not exceed 10% of their arithmetic mean.

## **Appendix B**

**(Normative appendix)**

**Detection of cell markers (Flow cytometry)**

**B.1 Instruments and equipment**

B.1.1 Flow cytometer.

B.1.2 Bench-top centrifuge.

B.1.3 Electronic balance.

**B.2 Reagents**

Unless otherwise stated, all the reagents used shall be analytical grade. The water used in the experiment shall be Grade 1 water as stipulated in GB/T 6682.

B.2.1 Phosphate buffered saline (PBS): pH7.4.

B.2.2 Paraformaldehyde (PFA): Purity 95%.

B.2.3 Bovine serum albumin (BSA): Purity≥98%.

B.2.4 Triton X-100.

B.2.5 Antibodies.

B.2.5 Use electronic balance (B.1.3), prepare the following solutions according to the relative requirements for flow cytometry: wash solution, fixing solution, blocking/permeabilization solution, antibody dilution solution.

**B.3 Sample storage**

The wash solution and fixed samples shall be stored at 2–8 ℃. The fixing solutions shall be aliquoted, sealed, labelled, and stored at below –20 ℃. Antibodies shall be stored according to the manufacturer’s instructions.

**B.4 Testing protocol**

B.4.1 Sample preparation and fixation

Collect samples by centrifuging single cell suspensions with Bench-top centrifuge (B.1.2) at 250 g for 3 min. Discard the supernatant. Resuspend the cells in an appropriate volume of fixing solution and incubate for 10 min in an ice bath. Wash the cell samples with an appropriate volume of wash solution for 3–5 times (3-5 min each time).

B.4.2 Blocking and permeabilization

Resuspend the fixed sample (B.4.1) with the blocking/permeabilization solution and aliquot the cells into two independent samples, which will be used as a testing sample and an isotype control sample respectively. Incubate on ice for 20 min, then wash the samples with the wash solution.

B.4.3 Antibody incubation.

Incubate the samples with the diluted antibodies or corresponding isotype controls according to the manufacturer’s instructions.

B.4.4 Filtering and loading

Resupend the samples with wash solution and then transfer the cell suspension into flow cytometry tube by filtering the samples through a mesh with 40 μm pores. Load the samples into the flow cytometer and perform testing according to the manufacturer’s instruction.

B.4.5 Gating

Gate the target population of cells based on particle size and transparency, excluding cell debris and other irrelevant particles. The gating of positive staining cells shall be determined by the fluorescence intensity using isotype controls as a reference. Both positive and negative experimental controls shall be set up for gating and the following analysis.

**B.5 Analysis of results**

Analyze the results using software according to manufacturer’s instructions.

## **APPENDIX C.**

**(NORMATIVE).**

**Detection of secretory function by enzyme-linked immunosorbent assay.**

**C.1 Instruments and equipment.**

C.1.1 Microplate Reader .

**C.2 Reagents.**

PEDF and VEGF ELISA kits.

**C.3 Sample storage.**

The supernatant of cell culture can be stored in the refrigerator at-20 ℃.. ELISA kits should be kept in accordance with the instructions.

**C.4 Testing protocol.**

Follow the kit instructions and use the microplate reader (c.1.1) for testing.

**C.5 RESULT ANALYSIS.**

Calculated the test results by formula referring form instructions of the kit.
